# Withania somnifera in Women’s Hormonal Modulation: A Narrative Review With Implications for Polycystic Ovary Syndrome and Premenstrual Syndrome

**DOI:** 10.7759/cureus.101431

**Published:** 2026-01-13

**Authors:** Maria Namysł, Simon Matczak, Sandra Dachowska, Jan Haraj, Ewa Majcherek, Marcin Sobkowiak, Maciej Bieniek, Kinga Woźniak

**Affiliations:** 1 Department of Medicine, Clinical University Hospital Poznan, Poznan, POL; 2 Department of Medicine, Poznan University of Medical Sciences, Poznan, POL; 3 Department of Medicine, Independent Public Healthcare Institution of the Ministry of the Interior and Administration in Poznań, Prof. Ludwik Bierkowski Hospital, Poznan, POL

**Keywords:** adaptogens, ashwagandha, fertility, polycystic ovary syndrome, premenstrual syndrome, withania somnifera

## Abstract

Background: Polycystic ovary syndrome (PCOS) and premenstrual syndrome (PMS) are among the most common hormonal diseases affecting women worldwide. Although multiple conventional treatments exist, they are often insufficient or poorly tolerated by the patients. *Withania somnifera *(WS) (ashwagandha) could provide a synergistic or alternative method of symptom management due to its endocrine, neuropsychiatric, and anti-inflammatory properties.

Aim of the study: This narrative review aims to evaluate the current evidence regarding the usefulness of *Withania somnifera* in aiding women’s hormonal balance, with a particular focus on its potential applications in PCOS and PMS.

Results: The current state of knowledge indicates that by modulating the hypothalamic-pituitary-adrenal (HPA) and hypothalamic-pituitary-gonadal axes, normalization of gonadotropins and estradiol levels is potentially achievable with *Withania somnifera*. This, along with a well-documented lowering effect on cortisol, improvements in metabolic markers, and the restoration of ovarian function in preclinical PCOS models, shows therapeutic potential. Crucially, ashwagandha’s impact on androgen levels appears sex-specific, as it does not seem to elevate testosterone in women, unlike in male study groups. In PMS, ashwagandha’s neuropsychiatric properties, most likely associated with GABAergic signaling and HPA modulation, may be beneficial in lowering fatigue, anxiety, and stress reactivity. Some studies also indicate possible analgesic activity of *Withania somnifera*.

Conclusions: Ashwagandha rises as a strong candidate for supportive treatment for managing the symptoms of PCOS and PMS by means of combined hormonal, metabolic, and neuropsychiatric effects. However, as current evidence relies heavily on preclinical models and extrapolation from other populations, further large-scale, randomized clinical trials using standardized extracts are necessary to establish definitive therapeutic protocols and confirm its clinical efficacy and safety.

## Introduction and background

Hormonal disorders, including polycystic ovary syndrome (PCOS) and premenstrual syndrome (PMS), impact the quality of life of women worldwide. PCOS is the most common endocrine disorder in women of reproductive age, with an estimated prevalence of 5%-20% in the female population. A key feature of this condition is excessive androgen production, mainly by the ovaries and, to a lesser extent, by the adrenal glands. The most common symptoms are acne, hirsutism, and menstrual irregularities. Notably, ovulatory dysfunction can lead to problems with fertility. Long-term complications include insulin resistance, type 2 diabetes mellitus, hypertension, and atherosclerosis [1].

PMS, on the other hand, is a condition that affects 10%-98% of women worldwide. It is defined by the occurrence of somatic or behavioral symptoms that start during the luteal phase and end within the first five days of menstruation. The most common symptoms include breast tenderness, bloating, headaches, and psychological manifestations such as mood liability, fatigue, anger, depression, anxiety, hypersomnia, or insomnia. To make the diagnosis, the symptoms must cause a significant impairment in the daily functioning of the patient and recur for at least two consecutive menstrual cycles [2,3].

Despite established treatment protocols, conventional therapies are often associated with adverse side effects and may be insufficient in fully alleviating the symptoms of these conditions. In PCOS, combined oral contraceptives are often first-line treatments to regulate cycles and reduce hyperandrogenic symptoms. However, they may cause headaches, nausea, and breast tenderness, and increase the risk of venous thromboembolism. Metformin, commonly used to improve metabolic parameters, is frequently associated with gastrointestinal distress, manifesting as abdominal discomfort, diarrhea, or dyspepsia. Antiandrogens can be effective for hirsutism and acne, yet their use is often limited by side effects such as menstrual irregularities, menopausal symptoms, or hyperkalemia [4]. In PMS, selective serotonin reuptake inhibitors (SSRIs) are effective for managing psychological symptoms, but their use may be associated with nausea, sleep disturbances, or sexual dysfunction, which often contribute to high discontinuation rates [5]. Hormonal interventions, including estrogens, anti-estrogens, androgens, and gonadotropin-releasing hormone agonists, may be related to menopausal symptoms and increased risk of osteoporosis [6].

The limitations of current therapeutic options have sparked interest in adaptogens as supportive treatments. Adaptogens are a class of non-toxic phytochemicals derived from plants that increase the organism’s resistance to stress and promote internal homeostasis. In clinical practice, adaptogens are most commonly investigated as supportive interventions for stress-related disorders, including chronic fatigue, anxiety, depression, and sleep disturbances. They are not considered disease-modifying therapies but are typically used as adjuncts aimed at symptom reduction and functional improvement [7].

Among these, *Withania somnifera* (WS), commonly referred to as ashwagandha, is one of the most commonly used [7,8]. WS, a perennial shrub of the Solanaceae family, has a well-documented history in traditional Ayurvedic medicine, where it has been used for over 2,500 years [9]. Current research confirms that ashwagandha exhibits a broad range of properties, including anti-stress, anti-inflammatory, antioxidative, antimicrobial, immunomodulatory, cardioprotective, neuroprotective, and endocrine-modulating effects [10,11]. Recent clinical trials and systematic reviews suggest that WS may modulate the secretion of cortisol and gonadotropins, thereby influencing steroidogenesis and sex hormone metabolism. While such effects have been documented in perimenopausal populations, their relevance to reproductive-age conditions characterized by hormonal dysregulation remains an area of growing interest. However, the current body of evidence is characterized by significant heterogeneity, with findings often dispersed across various preclinical models and human trials with diverse clinical endpoints that are not always directly comparable [12,13].

Accordingly, this narrative review aims to evaluate the therapeutic potential of WS in the management of PCOS and PMS, specifically through the lens of stabilizing the hypothalamic-pituitary-adrenal (HPA) and hypothalamic-pituitary-gonadal (HPG) axes. A narrative approach was adopted due to the substantial heterogeneity in study designs, populations, and extract standardizations, which currently precludes a formal quantitative meta-analysis. The analysis focuses on the normalization of serum cortisol, gonadotropin, and androgen levels, alongside the improvement of ovulatory function and the reduction of neuropsychiatric and physical symptoms. By integrating endocrine, metabolic, and clinical outcomes, this work highlights mechanistic insights, safety profiles, and current research gaps, providing a foundation for future targeted clinical trials.

## Review

Methodology

This report is structured as a narrative review. To enhance transparency and reproducibility, the literature search was conducted across PubMed, Web of Science, and Google Scholar using the following specific search strings: (“Withania somnifera” OR “ashwagandha”) AND (“PCOS” OR “polycystic ovary syndrome” OR “PMS” OR “premenstrual syndrome” OR “female hormones” OR “cortisol”).

Inclusion criteria focused on (1) randomized controlled trials (RCTs) involving women with endocrine or neuropsychiatric symptoms; (2) preclinical models (e.g., letrozole-induced PCOS rats) that provide mechanistic insights; and (3) systematic reviews on adaptogenic safety. Exclusion criteria included case reports, studies with poor methodological quality (as determined by lack of control groups or blinding in clinical settings), and non-English publications.

Botanical characteristics and phytochemical profile

WS is native to arid regions of South Asia, Mediterranean areas, the Canary Islands, and the Cape of Good Hope [12,14]. The plant’s fleshy roots contain the highest concentration of bioactive constituents. Compounds belonging to the withanolide class, mainly withanolide A, withanolide D, withaferin A, withanolide B, pubesenolide, and jaborosalactone D, have been identified as key contributors to the plant’s pharmacological effects [15]. Beyond withanolides, WS roots contain numerous phenolic acids, flavonoids, saponins, and glycosides. Studies have shown that these compounds exert synergistic activities that include, among many, anti-inflammatory, antioxidant, cytoprotective, and immunomodulatory effects [16].

Fundamental mechanisms of action of WS

Neuroendocrine Modulation

WS influences both the hypothalamic-pituitary-adrenal (HPA) and hypothalamic-pituitary-gonadal (HPG) axes, lowering serum cortisol levels [17,18]. These effects may involve direct inhibition of 11β-hydroxysteroid dehydrogenase type 1, the enzyme responsible for local cortisol regeneration, along with increased 5β-reductase activity, which results in more efficient cortisol clearance [19]. Moreover, it is suggested that ashwagandha modulates the secretion of corticotropin-releasing hormone and adrenocorticotropic hormone [20]. However, the precise mechanisms require further investigation [21].

GABAergic Signaling

Preclinical studies suggest that ashwagandha modulates the gamma-aminobutyric acid (GABA) pathway. It exerts agonistic activity at GABAρ1 and GABA-A receptors, which is suggested to contribute to the anxiolytic and sedative properties of the plant [21]. The main active ingredient responsible for this effect is triethylene glycol, which is mostly found in the leaves of the plant [22]. This GABAergic modulation is the key potential behind ashwagandha’s neuropsychiatric benefits.

Cellular and Molecular Effects

**Figure 1:**
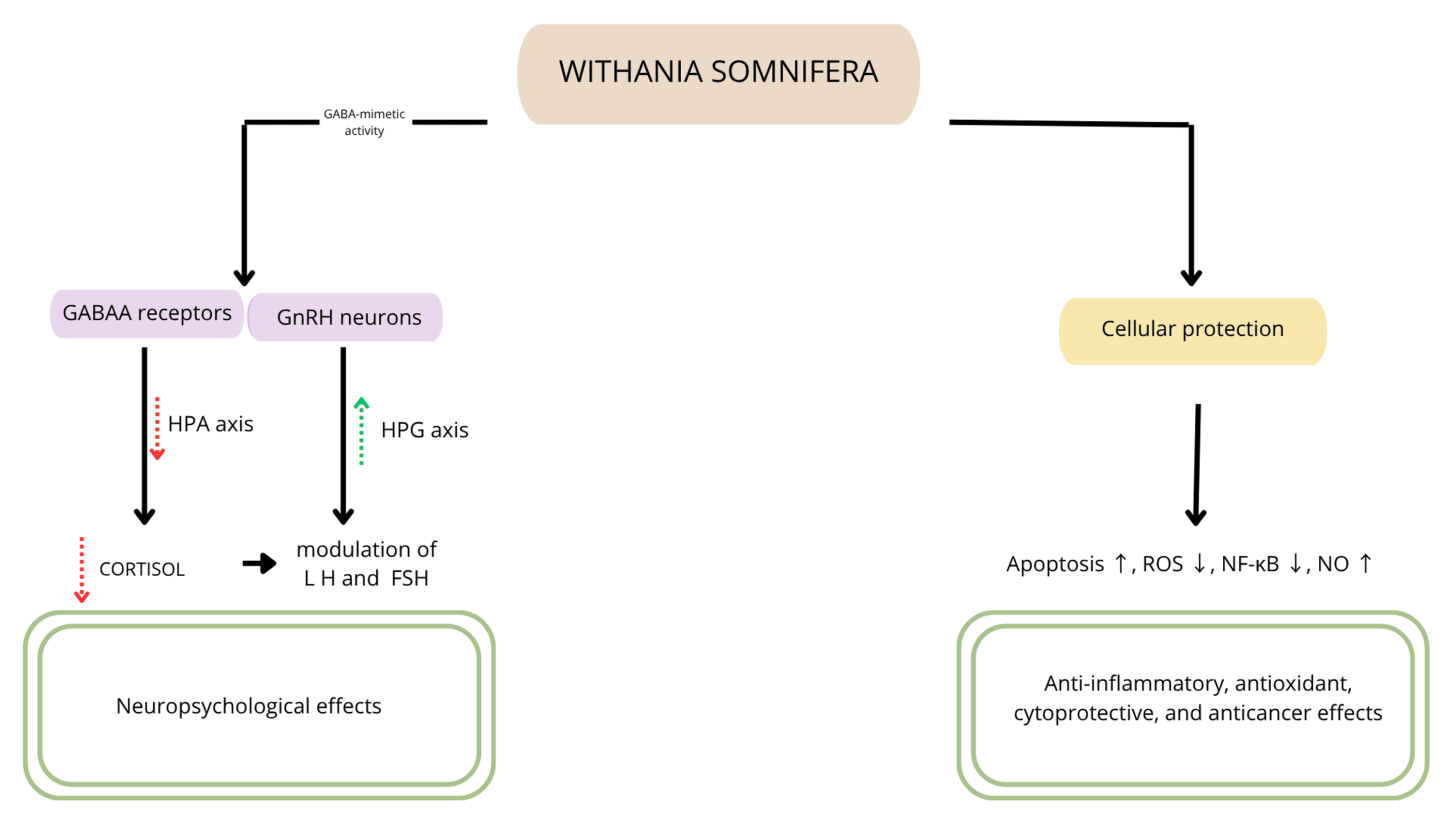
Fundamental mechanisms of action of WS GABA: gamma-aminobutyric acid, GnRH: gonadotropin-releasing hormone, HPA axis: hypothalamic-pituitary-adrenal axis, HPG axis: hypothalamic-pituitary-gonadal axis, LH: luteinizing hormone, FSH: follicle-stimulating hormone, ROS: reactive oxygen species, NF-κB: nuclear factor kappa-light-chain-enhancer of activated B cells, NO: nitric oxide, WS: *Withania somnifera*

In vitro studies indicate that ashwagandha’s withaferin A and withanolide A demonstrate cytoprotective and antioxidant effects. The mechanisms behind it most probably are the modulation of the apoptotic signaling and activating enzymes from the superoxide dismutase group [23]. Preclinical studies also suggest that withanolide A induces a vasodilatory response. This effect was observed in a study on rat aortic rings and was attributed to increased nitric oxide production [24]. Anti-inflammatory activity of WS involves the inhibition of mitogen-activated protein kinase (MAPK) and nuclear factor kappa-light-chain-enhancer of activated B cells (NF‑κB) signaling pathways. An in vitro study assessing the effect of ashwagandha root extract on skin cells has shown that it reduces the expression of inflammatory cytokines (e.g., interleukin-6 (IL-6) and tumor necrosis factor-alpha (TNF-α)) (Figure 1) [25,26].

Therapeutic potential of WS in PCOS

The pathophysiology of PCOS includes three models; however, they are not mutually exclusive. The first model points to dysfunction of the hypothalamic-pituitary axis and the subsequent excessive luteinizing hormone (LH) secretion leading to hormonal imbalance. The second model suggests that elevated androgen levels may result from hyperactivity of thecal cells in the ovary and insulin resistance with secondary hyperinsulinemia [27]. The third model states that hyperandrogenemia inhibits ovarian follicle development and ovulation, causing estrogen dominance over progesterone. This puts the patients at an increased risk of endometrial hyperplasia and endometrial cancer [28]. Current management of PCOS is largely symptomatic and focuses on hyperandrogenism and metabolic disturbances, reducing cardiovascular and cancer risk, and improving fertility outcomes [4]. According to the 2023 International Evidence-Based Guideline for PCOS, lifestyle interventions, hormonal therapies, and insulin-sensitizing agents remain the cornerstone of treatment. Herbal supplements, including WS, are not recommended due to insufficient high-quality clinical evidence [29]. The evidence discussed in the following sections is presented according to a hierarchy, beginning with available human clinical data, followed by indirect evidence from non-PCOS populations and preclinical models, and concluding with mechanistic interpretations.

Hormonal Effects

Direct clinical evidence supporting the use of WS for PCOS management is currently limited, but preclinical data and clinical studies conducted in non-PCOS populations demonstrate potential benefits. A randomized clinical trial that involved perimenopausal women supplemented with WS extract showed significant increases in estradiol levels along with decreases in LH and follicle-stimulating hormone (FSH) concentrations. The observed decrease in LH levels suggests that ashwagandha may help normalize the abnormal LH:FSH ratio typical in PCOS. Specifically, ashwagandha intake was associated with a statistically significant increase in serum estradiol and significant reductions in serum FSH and serum LH compared with placebo [13]. However, extrapolation of these findings to women with PCOS should be approached with caution. The hormonal effect of perimenopause, characterized by declining ovarian function and rising gonadotropins, is fundamentally different from that of PCOS, which is a condition affecting reproductive-age women defined by ovulatory dysfunction and hyperandrogenism. Nevertheless, the ability of ashwagandha to modulate gonadotropin levels warrants further research in this direction. A study targeted at patients with PCOS would help establish whether the normalization of the LH:FSH ratio corresponds with a clinically meaningful fertility rate.

In the preclinical studies using letrozole-induced PCOS animal models, data have shown over 85% similarity to human PCOS in hormonal profiles and ovarian histology, providing potential relevance. Treatment with the hydroalcoholic extract of WS combined with *Tribulus terrestris* successfully decreased testosterone and increased FSH levels after 28 days. Importantly, histopathological examination revealed improvements in ovarian morphology and increased presence of primary and secondary follicles, signifying improved ovarian function. A reduction in cyst formation was also noted [30]. However, the use of a polyherbal formulation constitutes a methodological limitation, as *Tribulus terrestris* itself has been independently studied for its effects on hormonal balance and ovarian function, thus creating a major confounder that limits definitive conclusions about ashwagandha’s specific role [31,32].

Gender-Specific Androgen Response

An important consideration in PCOS management involves the potential impact of WS on androgen levels. A randomized, double-blind, placebo-controlled study demonstrated that ashwagandha supplementation was associated with greater reductions in morning cortisol and dehydroepiandrosterone sulfate (DHEA-S) compared with placebo. Importantly, testosterone levels did not change significantly in females (0.2% reduction), which could be beneficial in PCOS, where a reduction in androgens is generally desired. Gender-wise analyses confirmed that cortisol and DHEA-S changes occurred in both men and women [18,33]. In contrast, clinical trials in men report testosterone increases of 10%-22% following WS supplementation, reflecting a sex-specific endocrine response likely mediated by WS’s modulation of the HPA axis [18,34,35].

However, in women, whose androgen production is more complex (involving ovaries and adrenal glands) and operates at much lower physiological levels, the same adaptogenic effect may not translate to a significant increase in circulating testosterone or may instead primarily modulate cortisol and DHEA-S levels. These observations highlight the sexual dimorphism in ashwagandha’s effect on androgen and suggest potential safety for use in PCOS [36]. Larger dedicated trials are needed to fully characterize the effects of WS on female hyperandrogenism. Such trials should include a comprehensive panel of androgens, including DHEA-S, androstenedione, and free testosterone, as well as sex hormone-binding globulin (SHBG), to completely understand the effects on the endocrine system.

Metabolic Effects

Ashwagandha may positively influence metabolic parameters often dysregulated in PCOS. Clinical studies conducted primarily in patients with no PCOS with type 2 diabetes or metabolic syndrome suggest that supplementation with WS may reduce lipid levels, body weight, and blood pressure, therefore reducing cardiovascular risk. In these trials, improvements were observed in total cholesterol, low-density lipoprotein (LDL) cholesterol, triglycerides, and increases in high-density lipoprotein (HDL) levels, as well as normalization of the levels of blood glucose, glycosylated hemoglobin (HbA1c), and insulin [37-40]. In a prospective, randomized, placebo-controlled study, healthy individuals receiving ashwagandha (330 mg or 500 mg daily for 28 days) showed significant decreases in mean systolic blood pressure during physical activity compared to placebo. While these findings suggest potential metabolic and cardiovascular benefits, they represent indirect evidence and cannot be directly extrapolated to women with PCOS [41,42].

Preclinical studies in animal and in vitro models provide insight into potential mechanisms underlying these effects. Withaferin A, a key active compound of WS, has been shown in vitro to increase glucose uptake in skeletal muscles [43]. The WS extract also inhibits preadipocyte differentiation and boosts energy expenditure through enhanced mitochondrial function in fat tissue and muscles, resulting in lower lipid levels [41]. Additional preclinical studies suggest anti-inflammatory effects in pancreatic tissue and anti-glycation activity [10].

Together, these data suggest a mechanistic rationale for the metabolic effects of WS, while highlighting the need for well-designed, PCOS-specific clinical trials to confirm its efficacy in this population (Table 1). 

**Table 1:** Mechanisms of action of WS and their implications for PCOS management HPA: hypothalamic-pituitary-adrenal, HPG: hypothalamic-pituitary-gonadal, WS: Withania somnifera, PCOS: polycystic ovary syndrome, CRH: corticotropin-releasing hormone, ACTH: adrenocorticotropic hormone, FSH: follicle-stimulating hormone, LH: luteinizing hormone, RCT: randomized controlled trial

Mechanism of action	Biological effects	Potential areas of research in PCOS	Sources
HPA axis modulation	Reduces cortisol levels [17-19]; potentially modulates the secretion of CRH and ACTH [20]	WS’s effect on insulin resistance and hyperandrogenism [18,33,36]	RCTs involving non-PCOS participants; preclinical data
HPG axis modulation	Inconclusive results: a study using WS combined with Tribulus terrestris reports a decrease in testosterone and an increase in FSH among PCOS-induced rats [30]; an RCT involving perimenopausal women describes an increase in estradiol levels and a decrease in LH and FSH concentrations [13]	WS’s effect on ovarian function; WS’s potential influence on normalizing the elevated LH/FSH ratio [13,30]	RCTs involving non-PCOS perimenopausal women; animal models with letrozole-induced PCOS
Hypoglycemic, hypolipemic, and anti-adipogenic activity	Increases glucose uptake and insulin secretion [43], increases mitochondrial activity in fat tissue in muscles [41]	WS’s effectiveness in reducing body weight and blood pressure, and minimizing the overall cardiovascular risk [37-40]	Clinical trials involving patients with metabolic syndrome; preclinical data

Therapeutic potential of WS in PMS

In PMS, the pathological fluctuations in progesterone and estrogen levels directly influence the activity of neurotransmitters such as serotonin and GABA. Allopregnanolone, progesterone’s metabolite, has a relatively weaker effect on the GABA activation in patients with PMS, which represents a key feature in this disorder’s neuroetiology. This phenomenon has been linked to mood disruptions and higher vulnerability to stress during the luteal phase [44,45]. Estrogen fluctuations in PMS decrease the availability of tryptophan, a precursor of serotonin, elevating the risk of anxiety and depression [46,47]. Moreover, in the luteal phase, enhanced reactivity of the HPA axis is observed, resulting in higher cortisol levels and disrupted nocturnal cortisol patterns in patients with PMS. Interestingly, this part of the cycle has been identified as the one in which PMS symptoms reach their peak intensity [44,48].

Stress Modulation

Clinical trials performed in patients under high stress demonstrate notable improvements in anxiety and mood symptoms following ashwagandha supplementation [18,20]. A randomized, double-blind, placebo-controlled trial involving 60 adults with self-reported high stress revealed a 23% drop in cortisol level after eight weeks of daily supplementation of ashwagandha in a dose of 240 mg. ​​This cortisol decrease correlated with a noticeable decline in anxiety levels using the Hamilton Anxiety Rating Scale compared to placebo groups [18]. In a larger clinical trial using higher doses of WS (600 mg daily), which involved 64 adults under chronic stress, a 30% cortisol reduction after 60 days was noted, along with similar improvements in all stress-related outcomes [49]. Another randomized, double-blind, placebo-controlled trial performed in 50 stressed adults found that ashwagandha intake significantly increased urinary serotonin levels and reduced the levels of morning salivary cortisol. Improvements in multitasking, concentration, and decision-making time were observed in the group administered WS compared to the placebo [20]. An important limitation of these trials is the relatively small study groups and the inability to directly translate these findings to PMS populations, given the hormonal pathophysiology of PMS-related mood disruptions.

A study on stress-induced rats observed a reduction in anxiety and depression-like symptoms in the group receiving ashwagandha root extract compared to the no-treatment group. This correlated with decreases in corticotropin (CRH), adrenocorticotropic hormone (ACTH), and cortisol levels [50]. Results from an animal study cannot be extrapolated to PMS populations due to inherently different physiology.

Preclinical studies suggest that the anxiolytic effect of ashwagandha is related to its ability to modulate the HPA axis and reduce cortisol levels [38]. Another prevalent theory states that ashwagandha’s withanolides increase tryptophan uptake, consequently improving serotonergic bioavailability at the central nervous system. The latter has been attributed to a lower risk of anxiety and depression, which could be potentially useful in PMS management [12,20]. Further research is necessary to define the exact mechanisms underlying this activity of WS and confirm its effectiveness in this area.

Sleep Quality Enhancement

Sleep disturbances are a common issue for patients with PMS, and ashwagandha has a well-documented impact on improving sleep parameters [34,51-54]. A randomized controlled clinical trial involving 144 healthy patients with no prior history of sleep disturbance showed that supplementation of WS in a dose of 120 mg a day for six weeks improved subjective sleep comfort in 72% of the trial participants, compared to 29% in the placebo group. Polysomnography performed in these patients revealed longer slow-wave patterns, suggesting a positive effect on the sleep quality of healthy adults. The complex pathophysiology of PMS necessitates further research on the effectiveness of WS in hormonal dysfunction-related sleep disturbances. A similar study performed by Langade et al. confirms these findings, as significant improvements in sleep onset latency, total sleep time, and subjective sleep quality were noted both in healthy participants and those with insomnia. Importantly, the sleep-promoting effect of WS was especially visible in the insomnia group [52]. Another trial involving healthy individuals who were administered 700 mg daily of ashwagandha for 30 days described that WS supplementation subjectively enhanced not only sleep quality but also energy and mental clarity [53]. These studies did not specifically target patients with PMS, but the results show insights into the effectiveness of WS supplementation in other applications. A potential explanation for these findings is WS’s effect on the GABA-A receptors, which are commonly targeted in insomnia treatment with conventional therapy [51,55].

Energy and Fatigue Management

Multiple clinical studies demonstrate ashwagandha’s efficacy in alleviating fatigue and improving subjective energy levels. A randomized controlled clinical trial in which 300 mg of ashwagandha was supplemented twice a day for eight weeks in the group of middle-aged adults with mild fatigue showed a significant fatigue reduction compared to placebo [33]. Additionally, a clinical study involving 18 people who were administered 1,250 mg of ashwagandha daily for 30 days demonstrated an improvement in muscle strength and physical performance [56]. No such research was conducted in PMS groups, leaving a potentially important gap in knowledge. The energy-enhancing effect of WS is largely unknown. It is probably mediated through multiple mechanisms, possibly by the HPA axis modulation that reduces glucocorticoid-induced metabolic suppression [38].

Anti-inflammatory and Analgesic Properties

The anti-inflammatory properties of ashwagandha could potentially be used to treat symptoms such as period pain, breast tenderness, or headaches commonly affecting patients with PMS [25]. A randomized, double-blind, placebo-controlled clinical study involving 60 patients with knee joint pain revealed dose-dependent pain reduction following ashwagandha supplementation [57]. The aim of this study did not specifically target menstrual pain. The anti-inflammatory and analgesic mechanisms could suggest a potential for PMS-related pain symptoms, but such a conclusion would necessitate further studies in this application (Table 2).

**Table 2:** Mechanisms of action of WS and their implications for PCOS management HPA: hypothalamic-pituitary-adrenal, CRH: corticotropin-releasing hormone, NF-κB: nuclear factor kappa-light-chain-enhancer of activated B cells, MAPK: mitogen-activated protein kinase, IL-6: interleukin-6, TNF-α: tumor necrosis factor-alpha, IL-1β: interleukin 1β, SOD: superoxide dismutase, GABA: gamma-aminobutyric acid, REM: rapid eye movement, RCTs: randomized-controlled trials, PMS: premenstrual syndrome, PCOS: polycystic ovary syndrome

Mechanism of action	Biological effects	Potential areas of research in PMS	Sources
HPA axis modulation	Reduces cortisol levels [17,18]; modulates the secretion of CRH and ACTH [20]	Effect of WS on anxiety, irritability, and stress levels in patients with PMS [21,38,44,48,49]	Multiple non-targeted RCTs; preclinical data; no trials were conducted among patients with PMS
Anti-inflammatory activity (withanolides, withaferin A)	Inhibits NF-κB and MAPK; lowers the levels of IL-6, TNF-α, and IL-1β; elevates glutathione levels; increases SOD activity [24-26,54]	Utility of WS management of pain symptoms, such as headaches, breast tenderness, and abdominal discomfort [25,57]	In vitro studies; trials on animal models
Serotoninergic activity	Enhances tryptophan availability [20]	Effect of WS on depressive symptoms and mood lability [18,20,50]	RCTs in the healthy population, no trials in patients with PMS
Sleep-promoting activity	Modulates the activity at GABA-A receptor levels, mainly via triethylene glycol [22,49]	Effect of WS on sleep quality, improvement of sleep comfort, and REM sleep quality; effects on mental clarity, reduction of fatigue, and stress levels in patients with PMS [31,51-54]	RCTs in the healthy population, no trials in patients with PMS

Safety profile of WS

Toxicity studies of whole plant extracts and isolated components of WS portray ashwagandha as a generally safe compound. Randomized controlled trials demonstrate that high-concentration WS root extract is well-tolerated, with adverse effects comparable to placebo groups. Side effects reported by the trials are usually mild, with the most prevalent being drowsiness, gastrointestinal upset, or headache in less than 5% of participants [49,58,59].

However, a comprehensive safety assessment must be implemented in certain populations. Due to WS’s ability to stimulate the immune system by increasing lymphocyte activity, caution is advised for individuals with autoimmune conditions, as the herb could exacerbate disease activity. Furthermore, ashwagandha has been documented to increase thyroid hormone levels (T3 and T4) in some studies. While this effect may be beneficial for patients with subclinical hypothyroidism, individuals with thyroid disease, particularly autoimmune thyroid disorders or those receiving thyroid hormone replacement, require close biochemical monitoring to avoid hormone excess or loss of disease control [60].

WS use during pregnancy requires caution due to limited safety data in this group. Traditionally, high doses were considered to have abortifacient properties, and while modern standardized extracts have not been shown to be teratogenic in animal studies, their use during pregnancy should be considered contraindicated until human safety data are available [61,62].

Drug interactions appear to be minimal; however, potential interactions with sedative medications need to be taken into account due to possible additive central nervous system depressant effects [63,64]. This includes benzodiazepines, barbiturates, and other CNS depressants. Similarly, due to its potential hypoglycemic and hypotensive effects, patients taking antidiabetic or antihypertensive medications should monitor their parameters, and dosages may need to be adjusted to prevent adverse effects [38]. Data on lactation safety are similarly lacking, and use during breastfeeding is not recommended. No published clinical studies have evaluated the excretion of *Withania somnifera* components into human breastmilk or the safety and efficacy of its use by nursing mothers and infants, and therefore, its use during lactation should be avoided [65].

Although randomized trials have not identified significant hepatotoxicity, increasing clinical evidence indicates that *Withania somnifera* can rarely cause drug-induced liver injury. A case series from Iceland and the US Drug-Induced Liver Injury Network described five patients with cholestatic or mixed liver injury occurring 2-12 weeks after ashwagandha exposure, with clinical resolution after discontinuation [66]. More recently, a multicenter Indian series reported predominantly cholestatic hepatitis linked to single-ingredient ashwagandha products, with severe outcomes including acute-on-chronic liver failure in patients with pre-existing liver disease [67]. These findings suggest that, while uncommon, ashwagandha-associated hepatotoxicity may be clinically significant, particularly in vulnerable populations.

Limitations

Existing research on *Withania somnifera* (WS, ashwagandha) in the context of PCOS and PMS is promising but currently constrained by several important limitations. As a narrative review, this article does not aim to exhaustively capture all available studies but rather to critically integrate mechanistically and clinically relevant evidence. The primary limitation of this review is the strong reliance on preclinical studies and animal models. While letrozole-induced PCOS models in animals provide valuable insights due to their high degree of resemblance to human PCOS, the translation of findings from rodent models to human pathophysiology must be approached with caution due to inherent species differences in steroidogenesis and reproductive endocrinology.

The second limitation is that high-quality randomized controlled trials evaluating the effects of WS in women with PCOS or PMS are notably limited in number and scope. The clinical evidence presented on WS use in PMS is extrapolated from studies conducted on other populations, primarily individuals with chronic stress or general anxiety. While the underlying mechanisms (e.g., HPA axis dysregulation and GABAergic tone) are relevant to PMS pathophysiology, the cyclical nature of PMS symptoms presents a unique clinical and biological context. The implications for WS use in PCOS, on the other hand, largely come from human trials involving perimenopausal women. The etiology of perimenopause is fundamentally different from disturbances present in women of reproductive age with PCOS, thus constraining the generalizability of outcomes regarding LH:FSH normalization and estradiol modulation.

The third limitation is that considerable heterogeneity exists in dosing regimens, extract standardization, treatment durations, and outcome measures across the studies reviewed. Many trials use different WS preparations, which may vary in withanolide content, purity, or presence of other bioactives, leading to inconsistencies in pharmacological activity and clinical effect size. There is also a lack of consensus regarding optimal WS dosing for hormonal versus neuropsychiatric outcomes, and long-term safety data for these populations remains sparse.

The fourth limitation is that gender-specific responses to ashwagandha have been documented, particularly with respect to androgen modulation. While WS appears to elevate testosterone in men with suboptimal baseline levels, no significant androgen change is consistently observed among supplemented women, which might be desirable in the context of PCOS. This warrants more specific profiling of androgen indices, including DHEA-S, androstenedione, and SHBG, in larger randomized controlled trials.

Future well-designed randomized trials and standardized outcome reporting will be necessary to enable quantitative statistical synthesis, including meta-analytical approaches.

Future directions for research

To address these gaps, future research should involve larger randomized controlled trials specifically targeted at women diagnosed with PCOS or PMS. Studies should employ standardized, pharmacologically characterized WS extracts with known withanolide concentrations to allow reproducibility and inter-study comparison. Comparative effectiveness research directly measuring clinical outcomes, such as rates of menstrual normalization, ovulation, live birth, and validated symptom reduction in PMS, is urgently needed. Future studies should include comprehensive endocrine panels, including all major androgens and SHBG, to better elucidate the nuanced effects of WS across diverse hormonal backgrounds. Given the observed gender-specific effects, research should incorporate sex-stratified analyses, as well as subgroup analyses by PCOS or PMS phenotype, age, and comorbid metabolic or psychiatric disorders.

## Conclusions

Preclinical data and non-targeted clinical trials suggest that WS has significant therapeutic potential considering its multi-target modulation of the HPA and HPG axes, GABAergic signaling, and anti-inflammatory pathways. Its cortisol-lowering properties may specifically counteract insulin resistance in PCOS while mitigating anxiety, stress, and sleep disturbances in patients with PMS. Its ​​anti-inflammatory effect, on the other hand, has the potential to reduce pain symptoms present in PMS. In PCOS cohorts, WS may additionally aid in normalizing the LH:FSH ratio and improving overall ovarian function. The suspected lack of influence on androgen levels in women appears beneficial for patients with PCOS, although this gender-specific androgen response requires confirmation by further investigation.

However, despite these promising insights, the clinical integration of WS into the management of PCOS and PMS is currently limited by a lack of targeted human trials and significant heterogeneity in extract standardization. The existing evidence relies heavily on preclinical models and perimenopausal cohorts, which necessitates a cautious approach when extrapolating findings to reproductive-age populations. To address these gaps, large-scale randomized controlled trials specifically targeting PCOS and PMS populations are essential. Future research must use standardized, pharmacologically characterized extracts and include comprehensive endocrine panels to assess clinical outcomes such as ovulation rates and menstrual regularity. Investigating the long-term safety and optimal dosing regimens remains essential for the formal inclusion of WS in clinical practice.
